# The co-injection of antioxidants with foot-and-mouth disease vaccination altered growth performance and blood parameters of finishing Holstein steers

**DOI:** 10.5713/ajas.18.0609

**Published:** 2018-10-29

**Authors:** Jakyeom Seo, Minho Song, Namchul Jo, Woonsu Kim, Sinyong Jeong, Jongnam Kim, Seyoung Lee, Seongwon Seo

**Affiliations:** 1 Division of Animal and Dairy Sciences, Chungnam National University, Daejeon 34134, Korea; 2 Life and Industry Convergence Research Institute, Department of Animal Science, Pusan National University, Miryang 50463, Korea; 3 Department of Food Science & Nutrition, Dongseo University, Busan 47011, Korea; 4 Division of Animal Husbandry, Yonam College, Cheonan, 31005, Korea

**Keywords:** Holstein Finishing Steer, Foot-and-mouth Disease, Vaccination, Antioxidants, Animal Health

## Abstract

**Objective:**

This study was conducted to evaluate whether the co-injection of antioxidants together with foot-and-mouth disease (FMD) vaccination has the potential to attenuate the negative effects caused by vaccination in Holstein finishing steers.

**Methods:**

A total of 36 finishing Holstein steers (body weight [BW]: 608±45.6 kg, 17 months old) were randomly allocated to one of three treatments: i) control (CON, only FMD vaccination without any co-injection), ii) co-injection of commercial non-steroidal anti-inflammatory drugs (NSAID) with FMD vaccination at a ratio of 10:1 (NSAID vol/FMD vaccine vol) as a positive control (PCON), iii) co-injection of commercial mixture of vitamin E and selenium with FMD vaccination (VITESEL) (1 mL of FMD vaccine+1 mL of antioxidants per 90 kg of BW). Changes in growth performance and blood parameters because of treatments were determined.

**Results:**

No significant difference in BW, average daily gain, and dry matter intake of the steers was observed among the treatments. The FMD vaccination significantly increased white blood cells (WBC), neutrophils, platelets, and mean platelet volume (p<0.01) in blood analysis. The count of lymphocyte tended to increase after vaccination (p = 0.08). In blood analysis, steers in VITESEL tended to have higher numbers of WBC, neutrophils, and platelets compared to that of other treatments (p = 0.09, 0.06, and 0.09, respectively). Eosinophils in VITESEL were higher than those in PCON (p<0.01). Among blood metabolites, blood urea nitrogen and aspartate transaminase were significantly increased, but cholesterol, alanine transferase, inorganic phosphorus, Mg, and albumin were decreased after FMD vaccination (p<0.01).

**Conclusion:**

The use of antioxidants in FMD vaccination did not attenuate growth disturbance because of FMD vaccination. The metabolic changes induced by vaccination were not controlled by the administration of antioxidants. The protective function of antioxidants was effective mainly on the cell counts of leukocytes.

## INTRODUCTION

Foot-and-mouth disease (FMD) is a highly contagious viral disease capable of affecting domestic cloven-hoofed animals, including swine, sheep, cattle, and goats, but also wild animals, such as deer [1]. Once infected, the animals exhibit fever, lameness, and vesicular forms on the snout, feet, tongue, and teats [2].

The government in South Korea decided to massively cull animals infected or suspected be to be, after the first report of the emergence of FMD in November 2010, but failed to prevent the spread of FMD throughout the entire region. Consequently, resident farmers raising cattle, goats, and pigs can not sell their animals or animal products without a certification of FMD vaccination, which is a mandatory regulation constituted by the South Korea government because of the widespread outbreak of FMD from November 2010 to March 2011 in South Korea. Although vaccination strategy against FMD is the strongly recommended way to reduce the spread of the disease, there are several negative effects should be considered, including the stressful management required to inject the FMD vaccine, followed by a possible inflammatory response and loss of productivity. Yeruham et al [3] reported that the vaccination against FMD could negatively affect growth, pregnancy, and milk production in ruminants.

Thus, a way to ameliorate the negative effect caused by the FMD vaccination is needed; however, to our knowledge, such studies have focused on developing more suitable vaccines for specific local conditions [4], rather than finding appropriate materials that may reduce harmful effects after the FMD vaccination in ruminants. Previously, colleagues, Jo et al [5] tested non-steroidal anti-inflammatory drugs (NSAID) and γ-aminobutyric acid with respect to the vaccination of FMD in castrated goats, and indicated that the use of NSAIDs can attenuate the negative effects caused by the FMD vaccination mainly because of the function of NSAIDs, which can inhibit cellular inflammation. Because the inflammatory response is the adaptive mechanism of the inherent immune system to infected or damaged sites [6], the vaccine challenge can also induce inflammation [7] and this response has to be terminated as quickly as possible; otherwise, the chronic inflammation is associated with dysregulated and harmful conditions, thereby inducing complicated disorders [7]. Vitamin E and selenium (Se) are known for antioxidants that can reduce the oxidative stress caused by reactive oxygen species (ROS), originating from the inflammatory response. Several studies evaluated the relationship between antioxidants and protection of immune cells, and demonstrated that antioxidants can protect lipid cellular membranes, as well as functional proteins of the immune cells, from oxidative damage by free radicals produced by inflammation [8–10].

However, to our knowledge, no studies have been conducted to evaluate the use of antioxidants with the FMD vaccination. We postulated that the application of antioxidants (vitamin E and Se) together with the FMD vaccination may attenuate the vaccine’s negative effects, thereby improving animal growth performance. Holstein finishing steers were used and not only their growth performance, but also blood cell counts were measured. We also investigated the changes in blood metabolites following FMD injection, which have not been investigated in previous studies.

## MATERIALS AND METHODS

This study was conducted at the Center for Animal Science Research, Chungnam National University, Korea. Animal use and the protocols for this experiment were reviewed and approved by the Chungnam National University Animal Research Ethics Committee (CNU-00301).

### Experimental design, animals, and diets

This experiment was performed for 4 weeks to evaluate the effects of a co-injection of antioxidants with the FMD vaccination on growth performance and blood indicators of Holstein steers. Before this study began, steers were separated into two groups and fed two different concentrates because all animals used in this study were involved in a different feeding trial. Because animals were fed two different concentrate mixtures because of a feeding trial, a randomized complete block design was used in this study. A total of 36 finishing Holstein steers (body weight [BW]: 608±45.6 kg, 17 months old) were randomly allocated to one of three treatments within 2 blocks by different concentrates: i) control (CON, only FMD vaccination without any co-injection), ii) co-injection of a commercial NSAID with FMD vaccination as a positive control (PCON), iii) co-injection of commercial mixture of vitamin E and Se with FMD vaccination (VITESEL). A commercial NSAID, dipyrone (Samyang Anipharm Co., Seoul, Korea) and mixture of vitamin E and Se (Merck Animal Health, USA) were used in this study. The commercial antioxidants contained 5 mg of Se and 50 mg of vitamin E per 1 mL. The FMD vaccine (Decivac FMD DOE; Intervet/Schering-Plough Animal Health, Boxmeer, Netherlands) was purchased and stored at 4°C until vaccination. Animals belonging to CON group were injected with 1 mL of the FMD vaccine intramuscularly behind the elbow according to the manufacturer’s instructions before the morning feeding on d 14 after starting the experiment. Animals in PCON acquired the same FMD vaccine mixed with a commercial NSAID (1:10 vol/vol) following the manufacturer’s protocol. Steers in VITESEL injected with mixture of FMD vaccine and commercial antioxidants (1 mL of FMD vaccine + 1 mL of antioxidants per 90 kg of BW) following the manufacturer’s protocol.

Two concentrates that were formulated to meet nutrient requirements for average daily gain (ADG) of 1,000 g/d [11], and ryegrass hay were offered to animals twice daily at 08:00 and 17:00 h. Diet formulation and chemical composition of the experimental diets are shown in Table 1. Additionally, steers had free access to drinking water throughout the experiment.

### Sample collection and measurements

Daily feed intake of each animal was recorded automatically (Dawoon Co, Incheon, Korea). Every weeks, the experimental diets were sampled, and BW of the animals was measured before the morning feeding at the start and end of the experiment. Diet samples were dried at 60°C for 96 h and ground through a cyclone mill (Foss, Hillerød, Denmark) fitted with a 1 mm screen. Then, they were stored at −20°C until chemical analysis [5]. Contents of dry matter (#930.15), crude protein (#990.03), ether extract (2003.05), acid detergent fiber (#973.18), and ash (#942.05) were determined as described by AOAC [12]. Lignin and neutral detergent fiber (aNDF) were analyzed using a heat stable amylase and expressed inclusive of residual ash, as described by Van Soest et al [13].

Blood samples were taken on the vaccination day just before vaccination (d0AM) and 6 h after vaccination (d0PM) to investigate hematological and metabolic response caused by the vaccination and treatments. To assess overall changes in blood, it was also sampled 14 days before (d-14 AM and d-14PM) and after vaccination (d14AM and d14PM) in the same manner as conducted on d 0. Therefore, a total of six blood samplings were performed (d-14AM, d-14PM, d0AM, d0PM, d14AM, and d14PM). Approximately 20 mL of blood was taken from the jugular vein of each steer and collected into a vacutainer tube containing ethylenediaminetetraacetic acid (EDTA) (Becton Dickinson Vacutainer Systems, Plymouth, UK), as well as a serum tube containing clot activator (BD Vacutainer; BD and Co., Franklin Lakes, NJ, USA). The EDTA tubes were placed on ice and then immediately transferred to the analytical laboratory of Animal Hospital at Chungnam National University for the complete blood count (CBC) analysis, which included white blood cells (WBC), lymphocytes, monocytes, eosinophils, neutrophils, the neutrophil to lymphocyte ratio (N:L), red blood cells (RBC), hemoglobin, hematocrit, platelets, and mean platelet volume (MPV). Serum was obtained by centrifugation at 1,300×*g* for 15 min at 4°C and frozen at −80°C until later analysis. The serum was analyzed for glucose, cholesterol, triglycerides, non-esterified fatty acid (NEFA), blood urea nitrogen (BUN), creatinine, aspartate transaminase (AST), alanine transaminase (ALT), Ca, inorganic phosphate (IP), Mg, albumin, and total protein using kits purchased from Wako Pure Chemical Industries, Ltd. (Osaka, Japan) and a clinical auto analyzer (Toshiba Acute Biochemical Analyzer-TBA-40FR, Toshiba Medical Instruments, Tokyo, Japan) following the procedures described by Wang et al [14].

### Statistical analysis

The data on growth performance were analyzed with PROC MIXED (SAS Institute, Cary, NC, USA). The fixed effects in the model were a co-injection treatment and the random effects were the animals within treatments.

The data on the CBC and blood metabolites were analyzed with PROC GLIMMIX (SAS Institute, USA). The fixed effects in the model included the co-injection treatment, blood collection, and their interaction. The orthogonal contrast was used to test the difference between before (d-14AM, d-14PM, and d0AM) and after (d-14AM, d-14PM, and d0AM) vaccination. Pair-wise comparisons of the least square means were conducted using the PDIFF option with the Tukey-Kramer adjustment. Statistical significance was declared at p<0.05, and a trend was discussed at 0.05≤p<0.1.

## RESULTS

### Growth performances

No significant difference in initial BW, final BW, ADG, concentrate dry matter intake (DMI), forage DMI, and total DMI of the steers was observed among treatments. However, co-injection of FMD vaccination with antioxidants tended to increase the feed conversion ratio (FCR) (p = 0.07, Table 2) compared to that of CON.

### Blood cell and metabolites analysis

Among the items in the blood cell analysis, WBC, neutrophils, and platelets tended to be affected by treatments (p = 0.09, 0.06, and 0.09, respectively, Table 3). Steers in VITESEL tended to have higher WBC, neutrophils, and platelets compared to those in CON and PCON. Eosinophils in VITESEL were higher than those in PCON (p<0.01). Regarding the time of collection of blood samples, significant differences (p<0.01) in WBC, lymphocytes, neutrophils, the N:L, platelets, and MPV were observed. The N:L was highest at d-14AM (p<0.01). The FMD vaccination significantly increased (p<0.01) WBC, neutrophils, platelets, and MPV compared to that prior to vaccination. The count of lymphocytes after vaccination tended to be higher than that before vaccination (p = 0.08).

No significant difference in any blood metabolites, except BUN and IP, was observed among treatments. Steers in PCON tended to have higher BUN (p = 0.06) compared to those in CON. Inorganic phosphorus in CON was higher (p<0.05) than that in VITESEL. However, a significant difference in blood metabolites was observed regarding blood collection time. Among those with significant differences, BUN and AST were significantly increased (p<0.01) but cholesterol, ALT, IP, Mg, and albumin were decreased (p<0.01) after FMD vaccination.

A significant difference in WBC, neutrophils, N:L, RBC, triglycerides, Ca, and IP, as well as a tendency in hematocrit and Mg, was observed among treatments at d0PM (Table 4). The WBCs and neutrophils in VITESEL were higher (p<0.01) than those in CON. The N:L in VITESEL was higher (p<0.01) than that in PCON. Hematocrit in VITESEL tended to be higher (p = 0.10) than that in CON. Steers in CON had higher RBC (p = 0.03) than that in PCON and higher Ca and IP (p = 0.04, p<0.01 respectively) than those of VITESEL. The concentration of triglycerides in PCON was higher (p<0.01) than that in all other treatments.

## DISCUSSION

This study was conducted to evaluate whether the co-injection of antioxidants together with the FMD vaccination had potential to attenuate the negative effects caused by vaccination in Holstein finishing steers. In ruminant nutrition, antioxidants mainly have been applied to periparturient dairy cattle undergoing huge metabolic stress because of calving, as well as feed changes in response to milk production [15,16]. During parturition, the production of prostaglandin F (during calving) and E (in response to pathogenic bacteria) in the uterus induces pro-inflammatory responses and contributes to the increased concentration of ROS in circulation [16,17]. The sharp increase of ROS during parturition may induce the imbalance between ROS and the antioxidants; therefore, the oxidative stress is driven. In such conditions, the representative antioxidants, vitamin E, and Se have been used not only to control the oxidative stress and immune response [15], but also as a biomarker to measure the degree of oxidative stress [16,18]. Although the mechanism of pro-inflammatory response induced by vaccination [7] in this study was different from those occurred during parturition in the above studies, we postulated that the capacity of antioxidants that reduce oxidative stress may also be helpful to vaccinated animals and improved growth performance would be possible [19].

Results from this study showed that the administration of vitamin E and Se followed by the FMD vaccination did not increase ADG and a significant difference in DMI (concentrate, forage, and total) was also not observed among treatments. Furthermore, steers in VITESEL tended to have greater FCR than those in CON. In ruminants, it is known that vaccinations against such viruses including FMD cause detrimental effects, such as a decrease in growth rate and milk production [5,20,21]. Brahman×Angus growing heifers (approximately 12 months old) in a vaccination group exhibited lower ADG compared to animals in a non-vaccination group without any reduction of DMI [20]. Although the experimental animals and the design differed from that of Arthington et al [20], the previous study was also conducted in our lab and reported a significant depression of ADG after the FMD vaccination in comparison to those before the vaccination was observed in growing castrated goats, except for those in the NSAID group [5]. The goats having a co-injection of NSAIDs with the FMD vaccination exhibited an increased growth rate. Although the direct interaction between the FMD vaccination and the change in growth performance could not be investigated in this study because of the absence of a non-vaccinated group, the results from our study suggested that the FMD vaccination did not compromise the growth rate compared to those a month prior (data not shown). One possible reason is that the severity of FMD and the side effects of the FMD vaccine is more harmful to young animals than those that are mature [2]. The animals used in this study were finishing steers (approximately 17 months old), which were more mature than animals used in previous studies; therefore, the negative effect of the FMD vaccination might have been attenuated.

Regarding the blood analysis (Table 3), the vaccination increased WBCs, neutrophils, lymphocytes, and platelets (p< 0.01, p = 0.08, p<0.01, and p<0.01, respectively) compared to those at the pre-vaccination stage, and a significant increase in WBCs and neutrophils was also observed between d0AM and d0PM. This results are consistent with the previous study that showed increases in the number of leukocytes and the N:L ratio after vaccination in ruminants [5], suggesting that the FMD vaccination induced a pro-inflammatory response. The significant increase in WBCs after the vaccination seemed to be primarily caused by the sharp increase in neutrophils (Table 3). Neutrophils are the first line of cell-mediated immune response against invading pathogens and use their phagocytic functions [22]. It is known that neutrophils account for 25% of leukocytes on average under normal conditions, but the number of neutrophils are dramatically increased during inflammatory challenge, accounting for up to 60% of leukocytes [22] in bovine. The proportion of neutrophils in WBCs after vaccination in this study increased from 48.1% (4.91 neutrophils/10.2 WBC at d0AM) to 57.4% (6.94/12.1 at d0PM) suggesting that the systemic inflammation induced by vaccination stimulated the increase in the number of neutrophils. Hence, steers in VITESEL had a higher number of leukocytes, including WBCs, eosinophils, and neutrophils compared to those in CON (Tables 3, 4). The important function of antioxidants regarding cells was to protect cellular lipids, proteins, and DNA from oxidation by ROS [15]. Because of their protective functions, studies reported that the supplementation in feeds or direct injection of vitamin E and Se increased not only phagocytic activity in neutrophils [10], but also the number of activated neutrophils [23]. The increase in platelet counts after vaccination from this study is agreement with a previous study [24], which reported that neonatal calves challenged with porcine *Hog* cholera vaccine exhibited an increase in platelet counts at 5 to 14 days post-vaccine challenge.

Blood metabolites were also measured to evaluate physiological changes in steers induced by different treatments and vaccination. No significant difference in glucose and NEFA after vaccination in this study suggested that energy metabolism in experimental animals was not changed by vaccination. Energy metabolism, inflammation, and immune function are closely linked with each other [17]; therefore, cows having a strong negative energy balance are associated with a more severe or prolonged inflammation [17]. The concentration of AST increased after vaccination (Table 3) indicating that vaccination may induce harmful effects to liver cells. Nath et al [25] reported that cross-bred cows affected with FMD had greater AST concentration compared to those in a control group suggesting that the effects of the FMD virus resulted in liver damage. Steers in VITESEL had a numerically higher AST concentration than those in CON, but a significant difference was not observed suggesting that the treatments could not protect liver function. Albumin is classified as a negative acute phase protein, which is dramatically decreased in inflammatory conditions [26]. Consequently, the concentration of total cholesterol is also classified as a negative biomarker during inflammation because lower lipid metabolism and cholesterol synthesis are commonly observed in a damaged liver during inflammatory conditions [16,25]. The decrease in albumin and total cholesterol after vaccination in this study suggested that the inflammatory response was prolonged to 14 d after vaccination and the administration of antioxidants might not control the inflammatory response. Spurlock [27] demonstrated that the consecutive inflammation induced by pro-inflammatory cytokines have been associated with altered nutrient utilization, and consequently, catabolic activities are amplified during inflammation. Because of that, it was speculated that the lower feed efficiency observed in steers in VITESEL compared to those in CON might be linked to prolonged inflammation.

## CONCLUSION

The present study was performed to evaluate whether the co-injection of antioxidants together with the FMD vaccination has the potential to attenuate the negative effects caused by vaccination in Holstein finishing steers. Results from this study demonstrated that the administration of vitamin E and Se followed by the FMD vaccination did not affect ADG and DMI, but FCR tended to be increased in VITESEL. The vaccination of FMD increased leukocytes, especially neutrophils, and lowered liver functions, which was indicated by several metabolites including cholesterol, AST, and albumin. The co-injection treatment with antioxidants only affected the number of leukocytes, indicating that the cell protective function of antioxidants might protect leukocytes from cell death. Overall, the use of antioxidants with FMD vaccinations did not attenuate the growth disturbance caused by FMD vaccination. The metabolic changes induced by vaccination were not controlled by the administration of antioxidants. The protective function of antioxidants was effective mainly on the cell counts of leukocytes.

## Figures and Tables

**Table 1:** Diet formulation and chemical composition (g/kg, DM basis, or as stated) of the experimental diets

Items	Concentrate A		Concentrate B	
Ingredients				
Corn, fine	45		337	
Corn, flaked	250		251	
Wheat, fine	242		117	
Wheat, flour	57		80	
Rice bran	47		27	
Palm oil	10		13	
Cottonseed, whole	34		34	
Cottonseed, hulls	18		-	
Rapeseed meal	-		54	
Soy hulls	113		-	
Corn gluten feed	90		-	
Molasses	55		45	
Cell mass lysine	13		8	
Urea	2		7	
Salt	2		2	
Limestone, fine	20		23	
Vitamin and mineral mix1)	2		2	
	
Chemical composition	**Concentrate A**	**Concentrate B**		**Rye grass, hay**
	
DM, g/kg as fed	867	865		927
CP	157	168		55
EE	47	47		8
Ash	55	60		75
NDF	201	168		770
ADF	71	70		462
Lignin	23	29		46

DM, dry matter; CP, crude protein; EE, ether extract; NDF, neutral detergent fiber; ADF, acid detergent fiber.

1) 33,330,000 IU/kg vitamin A, 40,000,000 IU/kg vitamin D, 20.86 IU/kg vitamin E, 20 mg/kg Cu, 90 mg/kg Mn, 100 mg/kg Zn, 250 mg/kg Fe, 0.4 mg/kg I, and 0.4 mg/kg Se.

**Table 2:** The effects of foot-and-mouth disease vaccination in different treatments on growth performance of Holstein finishing steers

Items	CON1)	PCON1)	VITESEL1)	SEM	p-value
Initial BW (kg)	608	608	608	5.5	1.00
Final BW (kg)	651	650	643	7.0	0.47
ADG (g/d)	1315	1272	1102	134.4	0.28
Concentrate DMI (kg/d)	9.21	9.52	8.79	0.460	0.31
Forage DMI (kg/d)	1.31	1.31	1.31	0.149	1.00
Total DMI (kg/d)	10.5	10.8	10.1	0.52	0.38
FCR2)	8.00^y^	8.96^xy^	9.56^x^	0.636	0.07

FMD, foot-and-mouth disease; SEM, standard error of the mean; BW, body weight; ADG, average daily gain; DMI, dry matter intake; FCR, feed conversion ratio.

1) CON, FMD vaccination with no treatments; PCON, FMD vaccination with injection of a non-steroidal anti-inflammatory drug; VITESEL, FMD vaccination with injection of antioxidants (a commercial mixture of vitamin E and Se).

2) DMI (kg/d)/ADG (kg/d).

x,y Means with different superscripts among treatments differ (0.05≤p<0.10).

**Table 3:** The effects of foot-and-mouth disease vaccination with different treatments on blood cell count and metabolites of Holstein finishing steers

Item	Treatments1)			Blood collection2)						SEM	p-value3)			
		
	CON	PCON	VITESEL	d-14AM	d-14PM	d0AM	d0PM	d14AM	d14PM		Treatments	Blood collection	Interaction	Before vs after vaccination
Blood cell analysis														
WBC (10^3^/μL)	9.27^y^	10.5^xy^	11.7^x^	9.01^c^	10.7^b^	10.2^bc^	12.1^a^	10.0b^c^	10.9^ab^	1.143	0.09	<0.01	0.81	<0.01
Lymphocytes (10^3^/μL)	3.77	4.75	4.53	3.19^b^	4.82^a^	4.54^a^	4.46^a^	4.23^a^	4.85^a^	0.611	0.26	<0.01	0.55	0.08
Monocytes (10^3^/μL)	0.11	0.13	0.11	0.11	0.11	0.16	0.06	0.16	0.10	0.051	0.91	0.30	0.51	0.43
Eosinophils (10^3^/μL)	0.53^ab^	0.43^b^	0.73^a^	0.51	0.52	0.56	0.68	0.49	0.61	0.128	<0.01	0.71	0.99	0.41
Neutrophils (10^3^/μL)	4.87^y^	5.13^xy^	6.33^x^	5.19^b^	5.21^b^	4.91^b^	6.94^a^	5.06^b^	5.35^b^	0.625	0.06	<0.01	0.08	<0.01
N:L	1.57	1.39	1.78	2.54^a^	1.27^b^	1.24^b^	1.82^ab^	1.31^b^	1.31^b^	0.328	0.37	<0.01	0.53	0.28
RBC (10^3^/μL)	7.59	6.98	7.19	7.59	7.12	7.01	7.96	6.95	6.91	0.498	0.44	0.21	0.79	0.91
Hemoglobin (g/dL)	9.98	10.1	10.3	10.7	10.1	10.1	10.2	9.86	9.90	0.339	0.62	0.13	0.83	0.15
Hematocrit (%)	31.3	31.8	32.4	33.6	32.4	31.5	31.5	31.2	31.0	1.05	0.56	0.12	0.71	0.27
Platelets (103/μL)	272^xy^	251^y^	310^x^	195^c^	268^b^	276^b^	268^b^	316^ab^	341^a^	26.5	0.09	<0.01	0.75	<0.01
MPV (fL)	3.83	3.88	3.67	3.81^bc^	3.77^c^	3.97^a^	3.68^cd^	3.93^ab^	3.61^d^	0.139	0.31	<0.01	0.77	<0.01
Blood metabolite analysis														
Glucose (mg/dL)	75.7	76.6	74.2	77.5^a^	73.3^b^	76.4^a^	78.4^a^	73.9^b^	73.5^b^	1.89	0.44	<0.01	0.30	0.35
Cholesterol (mg/dL)	113	120	110	120^a^	124^a^	110^b^	109^bc^	114b^c^	110^c^	9.8	0.58	<0.01	0.03	<0.01
Triglycerides (mg/dL)	13.0	15.7	14.9	15.1^a^	13.7^bc^	16.3^a^	12.9^c^	16.0^ab^	13.3^bc^	1.35	0.13	<0.01	<0.01	0.02
NEFA (mEq/L)	0.108	0.117	0.126	0.104^bc^	0.126^ab^	0.112abc	0.094^c^	0.135^a^	0.131^ab^	0.011	0.27	<0.01	0.78	0.30
BUN (mg/dL)	9.08^y^	10.7^x^	10.1^xy^	9.95^b^	10.8^a^	7.98^d^	8.71^c^	11.0^a^	11.2^a^	0.661	0.06	<0.01	0.02	<0.01
Creatinine (mg/dL)	1.30	1.29	1.27	1.29^ab^	1.29^a^	1.30^a^	1.31^a^	1.29^ab^	1.26^b^	0.048	0.83	<0.01	0.26	0.27
AST (IU/L)	63.7	61.0	60.5	59.6^ab^	52.3^b^	62.6^a^	66.8^a^	64.0^a^	64.9^a^	4.44	0.74	<0.01	0.09	<0.01
ALT (IU/L)	18.1	17.6	17.2	18.6^ab^	19.4^a^	16.5^c^	17.0^c^	17.6^bc^	16.7^c^	0.97	0.62	<0.01	0.29	<0.01
Ca (mg/dL)	10.4	10.3	10.2	10.2^bc^	10.1^c^	10.5^a^	10.2^bc^	10.4^ab^	10.2^bc^	0.09	0.23	<0.01	0.53	0.72
IP (mg/dL)	7.55^a^	7.25^ab^	7.12^b^	7.93^a^	7.91^a^	7.52^b^	6.73^c^	6.84^c^	6.93^c^	0.175	<0.05	<0.01	<0.01	<0.01
Mg (mg/dL)	2.23	2.30	2.18	2.37^b^	2.47^a^	2.22^c^	2.19^cd^	2.05^e^	2.13^de^	0.059	0.14	<0.01	1.00	<0.01
Albumin (g/dL)	3.42	3.46	3.47	3.49^ab^	3.54^a^	3.43^c^	3.46^bc^	3.42^c^	3.35^d^	0.040	0.41	<0.01	0.35	<0.01
Total protein (g/dL)	6.83	6.99	6.93	6.92^bc^	7.05^ab^	6.77^d^	6.81^cd^	7.08^a^	6.86^cd^	0.115	0.36	<0.01	0.60	0.86

SEM, standard error of the mean; FMD, foot-and-mouth disease; WBC, white blood cells; N:L, neutrophil to lymphocyte ratio; RBC, red blood cells; MPV, mean platelet volume; NEFA, non-esterified fatty acid; BUN, blood urea nitrogen; AST, aspartate transaminase; ALT, alanine transaminase; IP, inorganic phosphorus.

1) CON, FMD vaccination with no treatments; PCON, FMD vaccination with injection of a non-steroidal anti-inflammatory drug; VITESEL, FMD vaccination with injection of antioxidants (a commercial mixture of vitamin E and selenium).

2) d-14AM and PM, blood collection 14 days before vaccination at AM and PM; d0AM and PM, blood collection on the vaccination day at AM and PM; d14AM and PM, blood collection 14 days after vaccination at AM and PM.

3) Interaction; interaction between treatment and blood collection, before vs after vaccination; (d-14AM, d-14PM, and d0AM) vs (d0PM, d14AM, d14PM).

a,b,c,d,e Means with different superscripts among treatments or blood collection times differ (p<0.05).

x,y Means with different superscripts among treatments differ (0.05≤p<0.10).

**Table 4:** The effects of foot-and-mouth disease vaccination with different treatments on blood cell count and metabolites of Holstein finishing steers at d0PM

Items	CON1)	PCON1)	VITESEL1)	SEM	p-value
Blood cell analysis					
WBC (10^3^/μL)	10.6^b^	11.8^ab^	14.1^a^	1.35	0.03
Lymphocytes (10^3^/μL)	4.24	5.23	3.93	0.801	0.24
Monocytes (10^3^/μL)	0.058	0.067	0.042	0.095	0.97
Eosinophils (10^3^/μL)	0.675	0.517	0.833	0.222	0.36
Neutrophils (10^3^/μL)	5.58^b^	5.94^b^	9.28^a^	0.957	<0.01
N:L	1.41^ab^	1.26^b^	2.78^a^	0.586	<0.01
RBC (10^3^/μL)	9.36^a^	7.05^b^	7.47^ab^	0.920	0.03
Hemoglobin (g/dL)	9.68	10.3	10.7	0.631	0.25
Hematocrit (%)	30.4^y^	32.2^xy^	34.5^x^	1.94	0.10
Platelets (10^3^/μL)	277	248	279	39.8	0.68
Mean platelet volume (fL)	3.74	3.78	3.53	0.158	0.26
Blood metabolite analysis					
Glucose (mg/dL)	77.3	80.5	77.6	2.35	0.32
Cholesterol (mg/dL)	108	117	100	10.2	0.27
Triglycerides (mg/dL)	12.0^b^	16.5^a^	10.2^b^	1.73	<0.01
NEFA (mEq/L)	0.098	0.089	0.094	0.019	0.90
BUN (mg/dL)	8.15	9.35	8.63	0.764	0.29
Creatinine (mg/dL)	1.33	1.30	1.30	0.052	0.73
AST (IU/L)	68.5	59.9	72.0	6.25	0.14
ALT (IU/L)	18.3	16.3	16.3	1.30	0.20
Ca (mg/dL)	10.4^a^	10.3^ab^	10.0^b^	0.15	0.04
IP (mg/dL)	7.11^a^	7.08^a^	6.00^b^	0.254	<0.01
Mg (mg/dL)	2.18^xy^	2.28^x^	2.11^y^	0.075	0.10
Albumin (g/dL)	3.46	3.49	3.43	0.051	0.60
Total protein (g/dL)	6.78	6.94	6.72	0.137	0.25

SEM, standard error of the mean; WBC, white blood cells; N:L, neutrophil to lymphocyte ratio; RBC, red blood cells; NEFA, non-esterified fatty acid; BUN, blood urea nitrogen; AST, aspartate transaminase; ALT, alanine transaminase; IP, inorganic phosphorus; FMD, foot-and-mouth disease.

1) CON, FMD vaccination with no treatments; PCON, FMD vaccination with injection of a non-steroidal anti-inflammatory drug; VITESEL, FMD vaccination with injection of antioxidants (a commercial mixture of vitamin E and Se).

a,b Means with different superscripts among treatments differ (p<0.05)

x,y Means with different superscripts among treatments differ (0.05≤p<0.10).
